# Investigating the Relationship between Stable Personality Characteristics and Automatic Imitation

**DOI:** 10.1371/journal.pone.0129651

**Published:** 2015-06-16

**Authors:** Emily E. Butler, Robert Ward, Richard Ramsey

**Affiliations:** Wales Institute for Cognitive Neuroscience, School of Psychology, Bangor University, Adeilad Brigantia, Bangor, Gwynedd, Wales, United Kingdom; University of Bologna, ITALY

## Abstract

Automatic imitation is a cornerstone of nonverbal communication that fosters rapport between interaction partners. Recent research has suggested that stable dimensions of personality are antecedents to automatic imitation, but the empirical evidence linking imitation with personality traits is restricted to a few studies with modest sample sizes. Additionally, atypical imitation has been documented in autism spectrum disorders and schizophrenia, but the mechanisms underpinning these behavioural profiles remain unclear. Using a larger sample than prior studies (N=243), the current study tested whether performance on a computer-based automatic imitation task could be predicted by personality traits associated with social behaviour (extraversion and agreeableness) and with disorders of social cognition (autistic-like and schizotypal traits). Further personality traits (narcissism and empathy) were assessed in a subsample of participants (N=57). Multiple regression analyses showed that personality measures did not predict automatic imitation. In addition, using a similar analytical approach to prior studies, no differences in imitation performance emerged when only the highest and lowest 20 participants on each trait variable were compared. These data weaken support for the view that stable personality traits are antecedents to automatic imitation and that neural mechanisms thought to support automatic imitation, such as the mirror neuron system, are dysfunctional in autism spectrum disorders or schizophrenia. In sum, the impact that personality variables have on automatic imitation is less universal than initial reports suggest.

## Introduction

People automatically imitate the actions of those around them, a process that increases feelings of affiliation and rapport between interaction partners [1]. Although contextual or situational antecedents of imitation are becoming clearer [2], the underlying mechanisms remain poorly understood. It has been proposed that the mirror neuron system (MNS), a brain system that responds to performed and observed actions [3], is a key biological substrate for imitation [4]. Indeed, although contentious [5,6,7,8], a dysfunctional MNS has been implicated in atypical imitation abilities in autism spectrum disorders (ASD) and schizophrenia [9,10]. In addition, it has been suggested that enduring personality variables, such as empathy and narcissism, are antecedents to imitation [11,12]. However, there is limited evidence that demonstrates how personality influences automatic imitation. The aim of the current study is to investigate the relationship between automatic imitation and individual differences in stable personality and subclinical traits.

Different forms of imitation can be conceptually distinguished. Intentional imitation involves deliberately copying an observed movement, whereas automatic imitation refers to unintended copying behaviours [13,14]. In recent years, automatic imitation, which is also referred to as mimicry (e.g., [1]), has been studied through two main methodological approaches. The first approach typically involves a primary task, such as describing photographs, which is performed by a participant and a confederate. Whilst the primary task is performed, copying behaviours of the participant are covertly recorded. Using this approach, automatic imitation has been documented for a variety of behaviours, including foot shaking and facial expressions [11,15].

The second measure of automatic imitation is computer-based and uses a reaction time paradigm [13,16]. Similar to the first method, the measure of copying behaviour is incidental to the primary task. For example, the task may involve making a finger movement in response to a visual cue, whilst simultaneously observing another person’s finger movement [17]. If the observed finger movement is incongruent to the intended finger movement, a reaction time cost or interference is incurred. Such interference has been demonstrated to be a product of spatial and imitative components of the task [18]. Thus, interference reflects, in part, an urge to automatically copy an observed action, which must be inhibited when it is inconsistent with a motor intention [16]. Using these measures of automatic imitation, aspects of social context, such as pre-existing rapport, a goal to affiliate, mood, and emotional state have been shown to be antecedents of automatic imitation [2,13].

Insight into the antecedents of automatic imitation has also emerged by investigating how stable components of personality, rather than social context, may predispose some more than others to imitate more. Despite the wealth of knowledge regarding individual differences in personality [19–21], as yet, only a few studies have investigated how stable personality characteristics influence automatic imitation. For example, empathy, which includes emotional components such as emotional regulation, as well as cognitive components such as the ability to understand another person’s perspective and emotions [22], has been previously linked to automatic imitation using covert recording of copying behaviours [11]. More empathic individuals, as measured by a perspective-taking subscale, exhibited more copying behaviours than less empathic individuals. Additionally, narcissism, which is characterised by several behaviours including self-centeredness and a lack of empathy [23,24], has been linked with automatic imitation using the RT paradigm described above [12,25]. Participants with more narcissistic personality traits showed less interference from observing concurrent actions than participants with fewer narcissistic traits. Taken together, these results suggest that more self-interested individuals imitate less compared to those who are more interested in others.

While these studies provide tentative support for a relationship between stable personality traits and imitation, the evidence is not yet convincing. These studies had relatively small sample sizes (Empathy sample: n = 50; Narcissism samples: n = 18, n = 24), which represent the population mean less accurately than larger samples [26]. Furthermore, variables that may vary with narcissism or empathy and explain variance in imitative tendencies, such as overall latency in RT paradigms, age and sex, were not taken into account [27,28]. More generally, given recent failures to replicate landmark results in psychology [29,30], it is timely to investigate these initial findings more rigorously with larger sample sizes and by controlling for confounding variables [31].

Furthermore, as yet untested personality factors may also determine one’s tendency to imitate others. For example, using the reaction time measure of automatic imitation, simple sentences that prime a prosocial “state” have been shown to increase automatic imitation [32–35]. Consequently, stable traits that are associated with prosocial behaviours may also modulate the tendency to imitate others. Two of the Big-Five factors of personality, agreeableness and extraversion, have been associated with prosocial behaviours, including empathy [36], cooperative behaviour [37] and altruism [34]. In sum, due to a lack of evidence, the relationship between automatic imitation and stable personality characteristics is unclear.

Systematic variation in imitation abilities has also been discovered in atypical populations, such as ASD and schizophrenia, and many have argued that atypical imitation in these populations is underpinned by a dysfunctional MNS [9, 38–41]. While differences in intentional imitation may occur in ASD and schizophrenia [10,42], other research suggests that automatic imitation is intact, at least in individuals with ASD [43,44]. As such, the mechanisms that underpin differences in imitation abilities between typical and atypical groups are far from clear [5,6]. One way to gain insight into the cognitive mechanisms underpinning ASD and schizophrenia is by testing autistic-like and schizotypal traits in the typical population. It has been proposed that healthy participants show the same traits as those diagnosed with the disorder, but to a lesser extent [45]. Indeed, if autistic-like and schizotypal traits lie on a continuum from subclinical to clinical populations, and the workings of the MNS contribute to imitation difficulties present in ASD and schizophrenia, then a relationship between autistic-like or schizotypal traits and automatic imitation might be expected in the typical population.

The aim of the current study is to investigate the relationship between automatic imitation and individual differences in stable trait-based personality and subclinical traits. By doing so, we will provide deeper insights into the antecedents of automatic imitation. To assess automatic imitation, we use an established reaction time paradigm that provides an index of automatic imitative tendencies [17]. In addition, a series of questionnaires measure individual differences in personality (narcissism, empathy, and Big-Five factors) and subclinical (autistic-like and schizotypal) traits. In comparison to prior studies, by using a large sample, the data will reflect the population mean more accurately [26] and by using multiple regression analyses, we can control for variance in imitative tendencies explained by factors aside from our predictions [27,28], such as mean reaction time, sex, or age.

Two hypotheses can be distinguished. First, if stable personality factors are robust antecedents of automatic imitation, then relationships between imitation performance and personality variables should be observed [11,12,25]. Alternatively, if stable traits have little or no influence on automatic imitation, the view that enduring personality characteristics are universal antecedents of automatic imitation would require revision. Second, if atypical imitation abilities in ASD and schizophrenia are due to impaired engagement of the MNS [9,39–41] and a continuum exists between subclinical and clinical populations, the presence of autistic-like as well as schizotypal traits should result in reduced automatic imitation. By contrast, no relationship between automatic imitation and subclinical traits would provide evidence consistent with the view that the MNS is relatively spared in ASD and schizophrenia and other mechanisms may be responsible for atypical control of automatic imitation in these disorders [5,7,8].

## Method

### Participants

Two hundred and forty-three participants took part in this experiment for monetary compensation. All participants had normal or corrected-to-normal vision and provided written informed consent prior to data collection. Participants were excluded from the sample if accuracy (n = 8) or mean RT (n = 5) on the automatic imitation task was >3SD from the group mean. The final sample comprised 230 participants (133 female, 97 male; M_age_ = 21.62 years, SD = 5.41) who completed the personality, autism spectrum quotient, and schizotypy questionnaires described below. Fifty-seven of these participants (36 female, 21 male; M_age_ = 22.04 years, SD = 5.46) also completed a further two questionnaires, which measured empathy and narcisssm (see below for details). The data reported here were obtained from human participants under approval from the Research Ethics and Governance Committee of the School of Psychology at Bangor University. This approval does not include making the data available on a public repository. However, anonymised data used in this study can be requested from the corresponding authors.

### Materials and measures

#### Mini International Personality Item Pool (mini-IPIP)

The mini-IPIP [46] is a 20-item version of the International Personality Item Pool [47], which measures stable Big-Five personality traits. Particpants responded to statements that assess trait levels of extraversion (e.g., Talk to a lot of different people at parties), agreeableness (e.g., Sympathize with others’ feelings), conscientiousness (e.g., Get chores done right away), neuroticism (e.g., Have frequent mood swings) and intellect/imagination (e.g., Have a vivid imagination). Participants indicated on a 5-point scale, from very accurate to very inaccurate, how well each of the items describes them. This measure results in an average score between zero and five for each trait, with a score of five indicating high levels of the particular trait for each participant.

#### Short Autism Spectrum Quotient (AQ-10 Adult)

The AQ-10 [48] is a 10-item measure of autistic-like traits. Participants used a 4-point scale from definitely agree to definitely disagree to indicate how true each item is for them. Example items are “I find it difficult to work out people’s intentions” and “I often notice small sounds when others do not”. Participants scored one point per autistic-trait consistent answer, thus each participant scored between zero and ten, with 10 indicating high levels of autistic-like traits.

#### Brief Schizotypal Personality Questionnaire (SPQ-B)

The SPQ-B [49] is a 22-item questionnaire that assesses schizotypal personality disorder. A yes or no response is required to each statement or question. An example item is “Have you ever had the sense that some person or force is around you, even though you cannot see anyone?”. Participants scored one point per schizotypal consistent response and thus received a score from zero to twenty-two, with a score of twenty-two indicating a high number of schizotypal traits.

#### Narcissistic Personality Inventory (NPI-16)

The NPI-16 is a 16-item measure of narcissistic personality [50]. Participants indicated which statement out of a choice of two fitted them best and scored a point for every narcissism consistent statement that they chose. For example, participants could be presented with the following two statements; “I know that I am good because everybody keeps telling me so” and “When people compliment me I sometimes get embarrased”. In this example, they would score one point for choosing the first statement and zero for choosing the second statement. Seventeen participants refrained from answering one or more of the items in the NPI-16. The final score for each participant was the proportion of narcissism consistent responses that they gave, out of the total items that they answered. Therefore, scores were not influenced by the number of items that participants answered and ranged from zero to one, with one being more narcissistic.

#### Empathy Assessment Index (EAI-22)

The EAI-22 is a 22-item measure of empathic personaility [22]. Participants responded on a 6-point scale from never to always for items such as, “When I am with someone who gets sad news, I feel sad for a moment too”. Three participants refrained from answering one or more items on this measure. The final score for the EAI was also calculated as a proportion so that each participant’s score was not influenced by the number of items that they answered. To do this we calculated the maximum they could have scored if they gave 6 on every item that they answered. We then computed the proportion that their score was out of the maximum. Scores ranged from zero to one, with one being most empathic.

#### Automatic imitation task

**Fig 1:**
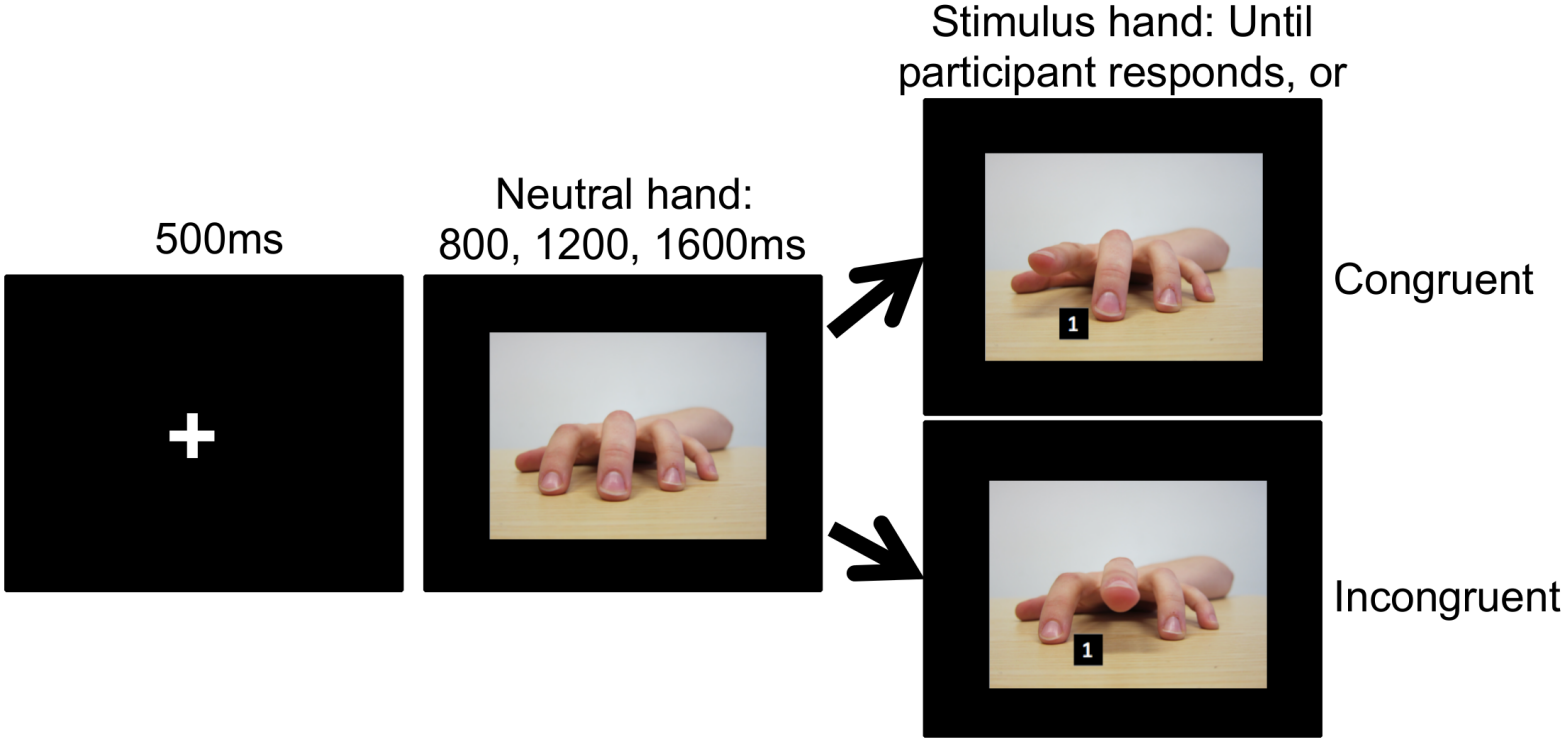
Automatic imitation task design. All trials for the automatic imitation task began with a fixation cross, then presentation of the neutral hand. After a variable inter-stimulus interval the target hand would be presented so the participant would be simultaneously cued to make their response, and view the hand appear to move.

The automatic imitation task was based on the paradigm developed by Brass and colleagues [17]. Prior to the start of the task, participants were instructed to hold down the ‘n’ key with the index finger of their right hand and the ‘m’ key with their middle finger. On each trial, upon presentation of the number target, instructions were to lift their index finger if it was a ‘1’ and their middle finger if it was a ‘2’, as quickly and as accurately as possible. The stimuli were images of a female left hand, viewed from the third-person so that the fingers of the hand extend towards the participants. The first image displayed the hand in a neutral position, resting on a flat surface. The other four images showed the hand with it’s index or middle finger lifted and a number ‘1’ or ‘2’ presented between it’s index and middle finger (Fig 1). Thus, there were 4 possible target trials and two conditions arose from these, congruent and incongruent. On congruent trials, the action that participants were cued to perform matched the action observed (e.g., the participant lifted their index finger whilst observing an index finger lift, or lifted their middle finger whilst observing a middle finger lift). On incongruent trials, the action that participants were cued to perform and the action observed did not match (e.g., the participant lifted their index finger whilst observing a middle finger lift, or lifted their middle finger whilst observing an index finger lift).

Each trial consisted of the presentation of a fixation cross for 500ms, followed by the neutral hand. The neutral hand remained on screen for a random interstimulus interval (ISI) of 800, 1200 or 1600ms before the target. The sucession of hand images produced apparent motion of a finger lifting at the same time that participants were presented with the number cue. The target display remained onscreen until participants’ made their response, but for no longer than 2000ms. Thus, total trial length varied, although it was never longer than 4100ms. Reaction time (RT) and error responses were recorded. RT was measured as the time from target onset to when participants lifted their finger from the ‘n’ or the ‘m’ key. Trials were considered correct if participants lifted the finger they were cued to lift. Trials were incorrect if they lifted the wrong finger, lifted a finger before target onset, or took longer than 2000ms to respond.

Trials were organised into a series of eight trials, which included two presentations of each trial-type in a random order. This eight-trial series was repeated until participants’ completed 60 trials. There should have been 64 trials per participants, but due to a technical error, the last four trials for each participant were not shown. However, the majority of participants (n = 147) completed 30 congruent and 30 incongruent trials. Ninety-two participants completed 30 trials per condition plus or minus one trial (e.g., 29 congruent and 31 incongruent). Four participants completed 30 trials per condition plus or minus two trials (e.g., 28 congruent and 32 incongruent). Pseudorandomisation permitted no more than 4 identical trials to occur consecutively.

Eight laptops were used to run the automatic imitation task, which was presented to participants using Psychophysics Toolbox, running in MATLAB. A 2 (congruency: congruent, incongruent) x 8 (laptop) ANOVA was used to test for differences in measured RT between the laptops. It was found that there was a main effect of congruency (F(1,222) = 1204.23, p<.001, η_p_^2^ = .844) such that participants were faster on congruent (M: 434.57, SE: 3.48) than incongruent (M: 519.59, SE: 4.65) trials. There was no main effect of laptop, (F(7,222) = 0.85,p = .550), and no interaction between congruency and laptop (F(7,222) = 0.74,p = .641). Therefore, for all analyses we collapsed data across the laptops used.

### Design and procedure

Participants first completed three questionnaires under no time constraint and in the following order: Mini-IPIP, Autism Quotient and Schizotypal Personality Questionnaire. As described above, whilst completing these questionnaires, participants could refrain from answering certain items if they wished to. As such, for each participant the number of items that contributes to each measure varies slightly. Participants then completed the automatic imitation task, including a 12-trial practice before the main task. The task was administered in groups of up to eight participants on laptops that were arranged in two rows with space between participants that were next to each other. Participants were monitored so that no interaction took place between them while they completed the task.

In a follow-up phase of the experiment, those participants from the first part who consented to be contacted regarding further participation were asked to complete measures of empathy and narcissism in an online survey. Their data was then linked back to data collected during the first part of the study. Out of the original sample, 57 participants completed two additional questionnaires, the Narcissistic Personality Inventory (NPI-16; [50]) and then the Empathy Assessment Index (EAI-22; [22]).

### Data analysis

#### Questionnaire coding

Questionnaires were scored as outlined above. Apart from participant sex, which was coded as -1 for males and +1 for females, raw scores on all of the questionnaires were centred (the group mean score for each variable was subtracted from each participant’s score on that variable; [51]). The AQ-10, SPQ-B and EAI-22 can all be broken down into component factors. The AQ-10 can be broken down into the factors of “attention to detail”, “attention switching”, “communication”, “imagination”, and “social”. The SPQ-B can be broken down into “cognitive-perceptual”, “interpersonal”, and “disorganised” factors. And finally, the EAI-22 can be broken down into “affective response”, “affective mentalising”, “self-other awareness”, “perspective-taking”, and “emotional regulation” factors. In all of the subsequent multiple regression analyses reported, total scores are used rather than component scores, except for the mini-IPIP as specific hypotheses regarded the subcomponents of extraversion and agreeableness. We ran these analyses with the AQ-10, SPQ-B and EAI-22 again with component scores, but this did not change the pattern of results for any of the measures. Cronbach’s alpha for extraversion (4 items; α = .77), agreeableness (4 items; α = .62), AQ-10 (10 items; α = .38), SPQ-B (22 items; α = .84), NPI-16 (16 items; α = .77), and EAI-22 (22 items; α = .89) were generally reasonably high.

#### Automatic imitation

Trials were removed if participants’ gave an incorrect response (7.23% of total trials), released a key during the ISI (0.20% of total trials) or were slower than 2000ms to respond (0.11% of total trials). Participants were then removed if mean RT (n = 5) or accuracy (n = 8) was >3SD from the group mean. For each participant mean average RT for congruent and incongruent trials was then calculated, as well as a congruency effect (incongruent RT minus congruent RT). Furthermore, accuracy was calculated as the percentage of the total trials on which participants gave the correct response.

#### Multiple regression analyses

In order to test our primary hypotheses, we used multiple regression analyses. Multiple regression tests whether predictor variables explain variance in a dependent measure (i.e., the congruency effect) whilst controlling for variance explained by other related factors [28]. As such, multiple regression can test whether or not individual differences in subclinical and/or stable personality factors predict the tendency to automatically imitate others, whilst controlling for the influence of other variables.

Before testing our predicitons, we constructed a base model. The base model was set up to account for factors that might significantly predict variance in participants’ congruency effect, which were not part of our primary hypotheses. We initially included mean RT (collapsed across congruent and incongruent trials), sex, and age in the base model (Table 1). Mean RT was included because we would expect participants with a faster RT to have a smaller congruency effect. In addition, research has shown that RT differs between sexes and across age groups [52]. In all subsequent tests of our hypotheses, any factors that significantly predict the congruency effect from the initial test of the base model were included.

**Table 1:** Summary table of the multiple regression models.

	Base model	Personality models			Subclinical models	
**Predictors**	Mean RT	Mean RT	Mean RT	Mean RT	Mean RT	Mean RT
	Participant Sex	Participant Sex	Participant Sex	Participant Sex	Participant Sex	Participant Sex
	Mean RT * Participant Sex	Extraversion	Narcissism	Empathy	Autism Quotient	Schizotypy
		Agreeableness				
		Conscientiousness				
		Neuroticism				
		Intellect/Imagination				

Summary of the models that will be tested using multiple regression to examine the predictive ability of each of the variables on the congruency effect.

To individually test each of our predictions, participants’ scores on predictor variables were added to the base model in separate hierarchical multiple regression models (Table 1). By doing so, any significant predictor would explain variance in the congruency effect, which is not already explained by the base model. Although it could be argued that the base model would explain a large amount of variance in the congruency effect and thus disadvantage the possibility of finding that a stable trait characteristic predicts automatic imitation, this approach avoids the possibility of finding that a trait predicts the congruency effect but that this relationship is in fact due to a third variable. However, in order to compare if any results did change when the base model was not inlcuded, we ran each regression model without the base model included. Finally, because sex differences have been recorded on trait factors [53], independent-samples t-tests were used to test for sex differences on each of the trait measures. For any measures that showed a sex difference, a sex*trait interaction term was calculated and assessed in separate muliple regression models. To calculate the interaction terms, continuous predictors were centered and categorical variables were dummy coded as negative one and positive one [51,54].

#### Group analyses

Comparisons between subgroups within our sample were performed in order to closely compare our results with prior work that has investigated similar questions (e.g., [12]). To do so, two groups were constructed based on each variable within the base model, as well as for each predictor variable. For sex, males (n = 97) and females (n = 133) were compared, and for all other variables the groups consisted of the highest or lowest scoring 20 participants. Congruency effects for high and low scoring groups on each variable were compared using independent-samples t-tests.

#### Power analyses

For the aforementioned analyses, we calculated statistical power using G*Power 3.1 [55]. The aim of these power analyses was to determine the effect sizes we had the power to detect with our samples of 230 and 57. In the primary test of our hypotheses, which involved multiple regression analyses, for the bigger sample (n = 230) we had power to detect large and medium effects, whereas in the subsample (n = 57) we had power to detect large effects and some medium effects.

## Results

### Questionnaires

Mean scores across the entire sample were calculated for each of the questionnaires. As sex differences have been found previously on trait measures of personality [53], independent-samples t-tests were implemented to examine whether there were sex differences on each of the stable trait characteristics in our dataset. Significant sex differences emerged on measures of agreeableness, neuroticism and intellect/imagination (all p’s <.05), such that female participants scored higher for agreeableness and neuroticism, and lower for intellect/imagination. There were marginally significant differences on conscientiousness, narcissism and schizotypy (all p’s between 0.05 and 0.1) such that female participants scored higher for conscientiousness, and lower for narcissism and schizotypy than male participants. No sex differences were found on extraversion, empathy and AQ (all p’s>0.7).

### Automatic imitation

As expected, participants were significantly more accurate (t(229) = 16.93,p<.001) and significantly faster (t(229) = -36.09,p<.001) on congruent compared to incongruent trials [17].

### Multiple regression

**Fig 2:**
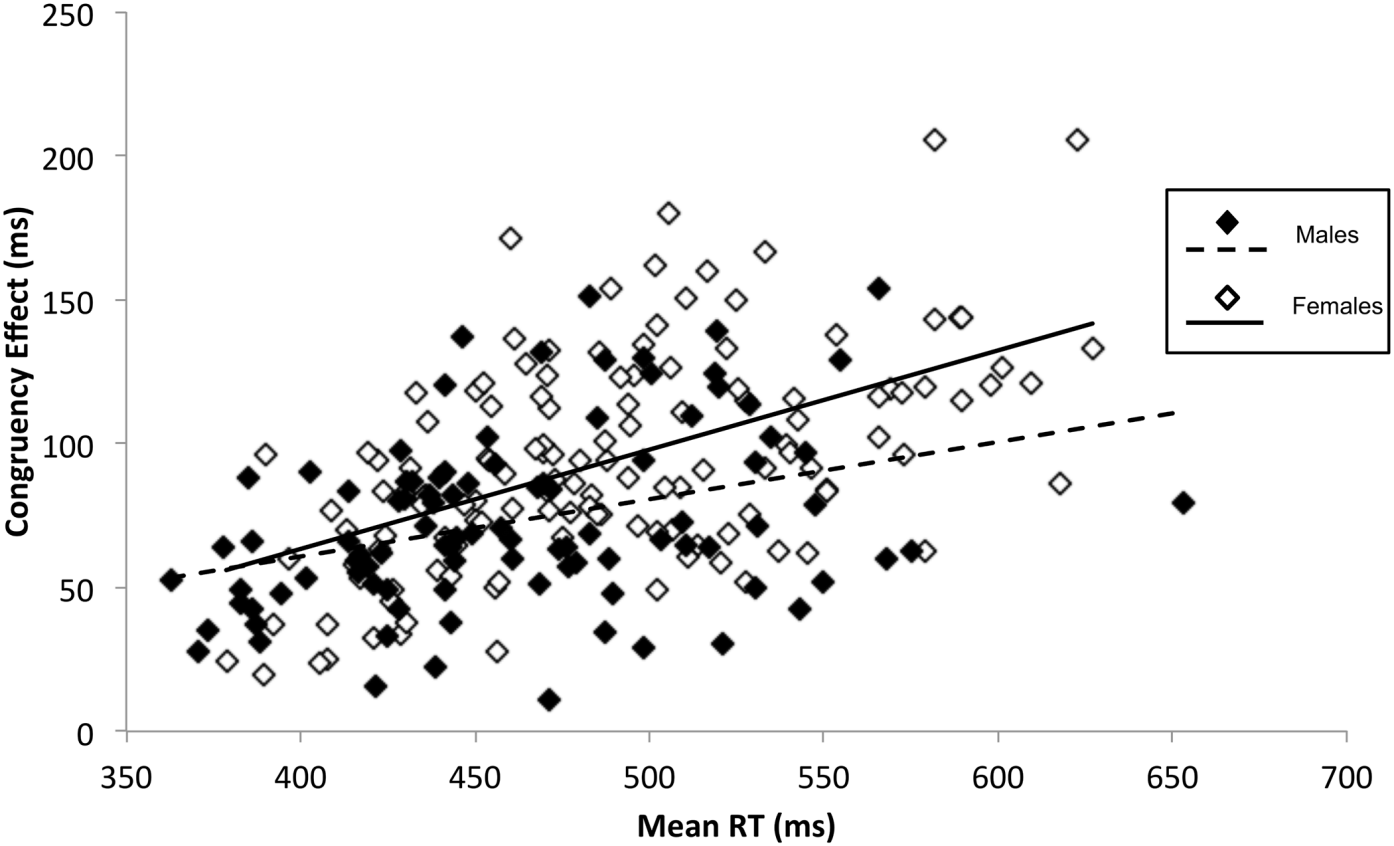
Sex and Mean RT difference in automatic imitation. The relationship between mean RT and congruency effect as a function of participant sex. Multiple regression analyses showed that mean RT, participant sex and the interaction between these two factors significantly predict the congruency effect. Individuals with a longer mean RT had a larger congruency effect than those with a shorter mean RT. In addition, women had a larger congruency effect than men. Furthermore, the interaction indicates that the sex difference was greater for individuals with longer than shorter RTs. Stable personality characteristics, including narcissism and empathy, as well as autistic-like or schizotypal traits, showed no reliable relationship with the congruency effect.

For transparency, simple correlations between the congruency effect and each predictor variable were calculated (S1 Table). However, as the goal of this study is to investigate the influence of predictor variables on the congruency effect, whilst controlling for confounding factors, we focus on the results from the multiple regression analyses. Results from all multiple regression models are summarised in Table 2. Results from the first base model showed that mean RT (B = 0.29, SEB = 0.04, t(225) = 7.79,p<.001) and sex (B = 6.70, SEB = 2.11, t(225) = 3.18,p = .002) significantly predicted variance in the congruency effect. The positive weighting for mean RT showed that as participants’ mean RT increased, their congruency effect increased. In addition, females had a larger congruency effect than males of about 13 ms. Age did not significantly predict the congruency effect (B = -0.16, SEB = 0.38, t(225) = -0.43, p = .668) and was thus removed from the base model in all further analyses. As both mean RT and sex predicted congruency effect, we also tested the mean RT by sex interaction term. The interaction between mean RT and sex was a significant predictor of the congruency effect (B = 0.07, SEB = 0.04, t(226) = 2.00, p = .047). As illustrated in Fig 2, the difference in congruency effect between men and women is greater at slower than faster mean RTs. The final base model (model 1) consists of mean RT, sex, and the mean RT by sex interaction.

**Table 2:** Results from the multiple regression analysis.

		Model 1 (n = 230): F(3,226) = 31.40, p<.001, R^2^ = .294				Model 2 (n = 224): F(8,215) = 11.33, p<.001, R^2^ = .296				Model 3 (n = 57): F(4,52) = 15.56, p<.001, R^2^ = 545				Model 4 (n = 57): F(4,52) = 15.72, p<.001, R^2^ = .547				Model 5 (n = 220): F(4,215) = 22.13, p<.001, R^2^ = .292				Model 6 (n = 199): F(4,194) = 19.83, p<.001, R^2^ = .290			
Predictors		B	SE B	t	p	B	SE B	t	p	B	SE B	t	p	B	SE B	t	p	B	SE B	t	p	B	SE B	t	p
**Base model predictors**	**Constant**	83.25	2.08	39.98	<.001	83.07	2.15	38.73	<.001	91.80	4.12	22.28	<.001	91.89	4.10	22.43	<.001	83.66	2.16	38.77	<.001	83.99	2.23	37.74	<.001
	**Mean RT**	0.27	0.04	7.45	<.001	0.27	0.04	6.83	<.001	0.50	0.07	6.81	<.001	0.50	0.07	6.88	<.001	0.27	0.04	7.15	<.001	0.27	0.04	6.73	<.001
	**Participant Sex**	6.98	2.08	3.35	.001	7.94	2.29	3.47	.001	-3.13	4.19	-0.75	.459	-3.06	4.09	-0.75	.458	6.91	2.16	3.20	.002	6.54	2.24	2.92	.004
	Mean RT * Sex	0.07	0.04	2.00	.047	0.08	0.04	2.07	.040	-0.05	0.07	-0.73	.470	-0.06	0.07	-0.80	.429	0.08	0.04	2.06	.040	0.08	0.04	2.15	.033
**Personality characteristic preditors**	**Extraversion**					2.46	2.44	1.01	.315																
	**Agreeableness**					-1.80	3.06	-0.59	.557																
	**Conscientiousness**					-2.79	2.41	-1.16	.249																
	**Neuroticism**					-1.16	2.62	-0.44	.660																
	**Intellect/Imagination**					0.57	3.19	0.18	.859																
	**Narcissism**									-2.67	20.52	-0.13	.897												
	**Empathy**													19.72	35.39	0.56	.580								
**Subclinical predictors**	**Autism Quotient**																	0.80	1.25	0.64	.522				
	**Schizotypy**																					-0.61	0.44	-1.39	.166

Model 1 includes the base model factors of mean RT, sex and the interaction between mean RT and sex. These base model factors predict the congruency effect but are not part of our personality-based and subclinical predictions. Across models 2–6, an additional trait factor is added to the base model in order to assess the influence of stable personality and subclinical traits on the congruency effect. By doing so, we are able to test whether personality variables influence performance on the automatic imitation task, in addition to factors within the base model.

After establishing the base model (model 1), subsequent models tested our main hypotheses. For each hypothesis, we created a model that included factors from the base model plus an additional predictor variable. By doing so, we were able to test whether any of our predcitor variables could explain variance in automatic imitation in addition to the variance explained by factors within the base model.

First, stable trait personality predictors were evaluated. Model 2, including the base model and all Big-Five factors, explained no more variance than model 1 (F(5,215) = 0.59, p = .705). In addition none of the Big-Five factors was a significant predictor on its own: extraversion (B = 2.46, SEB = 2.44, t(215) = 1.01, p = .315), agreeableness (B = -1.80, SEB = 3.06, t(215) = -0.59, p = .557), conscientiousness (B = -2.79, SEB = 2.41, t(215) = -1.16, p = .249), neuroticism (B = -1.16, SEB = 2.62, t(215) = -0.44, p = .660) and intellect/imagination (B = 0.57, SEB = 3.19, t(215) = 0.18, p = .859).

Two further personality measures, narcissism and empathy, were evaluated in a subsample of 57 participants. In two separate models, narcissism (model 3) and empathy (model 4) were added to the base model. Neither narcissim (F(1,52) = 0.02, p = .897; B = -2.67, SEB = 20.52, t(52) = -0.13, p = .897) nor empathy (F(1,52) = 0.31, p = .580; B = 19.72, SEB = 35.39, t(52) = 0.56, p = .580) significantly explained variance in the congruency effect in addition to the variance explained by the base model.

Second, subclinical predictors, including autistic-like and schizotypal traits, were evaluated in our larger sample (models 5 and 6). Neither autistic-like (F(1,215) = 0.41, p = .522; B = 0.80, SEB = 1.25, t(215) = 0.64, p = .522) nor schizotypal traits (F(1,194) = 1.93, p = .166; B = -0.61, SEB = 0.44, t(194) = -1.39, p = .166) significantly explained variance in the congruency effect in addition to variance explined by the base model.

Across models 1, 2, 5 and 6 (n = 230), mean RT and sex significantly explained variance in the congruency effect. In models 3 and 4 (n = 57) mean RT continued to predict the congruency effect, but sex did not (Table 2). However, in these multiple regression analsyes, the effect size for mean RT (f^2^ = 1.16) is considered large, whereas the effect size for sex (f^2^ = 0.06) is considered to be between a small and medium effect [56]. As a consequence, it is likely that a sample of 57 may only be large enough to detect the influence of mean RT and not sex, whereas the sample of 230 was large enough to detect both effects.

Due to prior research showing sex differences in basic trait measures [53], we assessed the possibilty that sex*trait interactions may explain variance in automatic imitation. Having established that sex differences emerged on certain trait varibales within our sample, we tested whether the relationship between sex and trait predicted performance on the automatic imitation task more than sex or trait variables alone. For those traits that showed significant, or marginally significant sex differences, additional multiple regression models were run to assess the predictive ability of interactions between sex and each trait on the congruency effect. Separate models were constructed to assess each sex*trait interaction. Each model included factors from the base model (mean RT, sex, and the mean RT by sex interaction), plus one trait predictor as well as the sex*trait interaction term. None of the sex*trait interactions significantly predicted the congruency effect on top of variance explained by the rest of the model.

Models 2–6 were also run not including the base model. This showed that no personality characteristic significantly predicted the congruency effect (all model p’s > .21), except the model including only schizotypy. As such, schizotypy did significantly predict the congruency effect (F(1,197) = 8.85, p = .029; B = -1.11, SEB = 0.50, t(197) = -2.20, p = .029) when factors within the base model were not controlled for.

### Group analyses

The regression approach above is an effective means for testing the effects of trait variables on automatic imitation. However, we also performed group analyses, comparing the congruency effects for the highest and lowest scorers on different stable traits. Group analyses rule out the possibility that, in our regression analyses, differences at the extremes might be washed out by variability in the middle. In addition, group analyses are consistent with the analytical approach taken in prior work and therefore make it easier to compare our results with prior studies that have not used multiple regression analyses. Except for sex, which was coded categorically, for each variable of interest, congruency effects for the 20 participants who scored the highest and the 20 who scored the lowest were calculated and compared. We investaged factors within the base model first. First, an independent-samples t-test showed that mean RT was significantly faster for the low than the high RT group (t(38) = -35.64, p<.001). Subsequently, an independent-samples t-test showed that groups that differed on mean RT showed a significant difference on conguency effect (Fig 2; t(38) = -6.81, p<.001). The high RT group showed a larger congruency effect than the low RT group. Splitting the data into groups based on sex also revealed a significant difference (t(228) = 4.51,p<.001,d = 0.61), such that females had a larger congruency effect than males. The results from this group analysis is therefore in agreement with the larger regression analysis.

Next we ran similar high-low group analyses for personality measures. First, to ensure that the high and low groups scored differently on the relevant personality characteristic, independent-samples t-tests were run. For all variables (each of the Big-Five factors, empathy, narcissism, AQ and schizotypy), scores on the relevant personality and subclinical measures were significantly different between the high and low scoring groups (all p’s < .001). Next, we ran the high-low group comparisons on congruency effects. We found no significant differences on congruency effects between those that scored high compared to low on each of the variables (all p’s > 0.156, except from agreeableness which was 0.072). Due to the smaller sample size for the narcissism and empathy measures, we also compared the top 10 to the bottom 10 scorers, and found the same pattern of results as with groups of 20.

## Discussion

Little is currently known about the relationship between automatic imitation and stable components of personality. The present study fails to support the view that inter-individual differences in stable personality and subclinical characteristics predict the extent to which individuals automatically imitate others. These findings suggest that automatic imitation is more resistant to variance in stable inter-individual differences than previously suggested [11,12,25]. Moreover, these data are consistent with the view that the MNS is relatively intact in ASD and schizophrenia and other mechanisms may be repsonsible for the imitative difficulties reported in these disorders [5,6,7,8]. Implications for understanding the antecedents and cognitive basis of automatic imitation are discussed below.

### Stable personality characteristics and automatic imitation

Contrary to our hypotheses, we found that trait personality constructs of narcissism, empathy, extraversion and agreeableness did not predict the extent to which participants automatically imitate. The lack of relationship between narcissism and automatic imitation is contrary to previous research [12,25]. Given recent failures to replicate landmark results in psychology [29,30], such conflicting results warrant close scrutiny. Indeed, it is important to note similarities and differences between this and prior work. In terms of similarities to prior work [12,25], we used the same automatic imitation task, the identical short-form measure of narcissism (NPI-16; [50]), and participants were comparable in age and predominantly female. Furthermore, to allow direct comparison with prior research we replicated the analytical approach used [12]. In comparison to these prior studies, the current work tested a larger sample (2–3 times larger) and used an additional analytical approach (multiple regression) that could control for potentially confounding variables [26–28,31,57].

Obhi and colleagues [12] showed that a group of high narcissists had a lower congruency effect than a group of low narcissists and the effect size was large (Cohen’s d = 1.02; [56]). Cohen’s d is typically used as a measure of effect size for group differences and is equal to the mean of group one minus the mean of group two, divided by the pooled standard deviation of the two groups. In this case, values of 0.2, 0.5, and 0.8 are generally considered to be small, medium, and large respectively [56]. We calculated Cohen’s d based on the methodological information and results provided by Obhi and colleagues [12]. Using the same analytical approach as used previously [12], which compared those who report high and low levels of narcissism, we show no differences in imitation performance between these groups. As such, we fail to replicate Obhi and colleagues finding in a sample that is 2–3 times larger.

Furthermore, using multiple regression we are able to control for additional and potentially confounding variables. Inspection of Obhi and colleagues [12] data shows that the high narcissism group were generally faster (mean RT = 477ms) than the low narcissism group (mean RT = 502ms). In the current study, regression analyses demonstrate a clear relationship between the congruency effect and mean RT, which has a large effect size [56]. To be consistent with the measure of effect size that we calculated for Obhi and colleagues [12], we used our group analyses to compute Cohen’s d as a measure of effect size for mean RT. We found that the difference in congruency effect between groups with low and high mean RT had a large effect size (Cohen’s d = 2.15). We do not, however, show a relationship between narcissism and the congruency effect. Even if we only regress narcissism and congruency effect, we show no relationship between these variables. As a consequence, it is likely that at least part, if not all, of the previously reported relationship between congruency effect and narcissism, could be explained by differences in mean RT. Our findings, therefore, suggest that the relationship between narcissism and automatic imitation is weaker than the initial evidence suggested. Further research that tests larger samples and controls for potentially confounding variables is required to further delineate the relationship between automatic imitation and narcissism.

Prior research has also demonstrated a link between empathy and automatic imitation by measuring copying behaviours during a live interaction between two people [11]. By contrast, we did not find any influence of empathy using a computer-based RT measure of automatic imitation. Therefore, the results of the current study suggest that the influence of empathic predispositions on automatic imitation is less universal than initially conceived. Indeed, contextual factors that are integral to live human interactions, such as emotion, may be needed to reveal relationships between empathy and automatic imitation. For example, research has shown greater automatic imitation of facial expressions by individuals who are more empathic than by those who are less empathic [58,59]. By contrast, in socially impoverished contexts, such as computer-based tasks, individual differences in empathy may have a reduced impact on automatic imitation. As such, future research should focus on identifying potential moderating variables in social cognition [60], such as emotion, as well as comparing different measures of automatic imitation, as it may help further delineate underlying mechanisms.

Automatic imitation has been shown to be sensitive to temporary social dynamics between interaction partners [2,13]. For example, priming of a prosocial state increases imitative tendencies in computer-based RT paradigms [32–35]. In the current study, we find no evidence that stable traits of extraversion and agreeableness, which are associated with prosocial tendencies [36–38], predict the tendency to imitate others. Automatic imitation may, therefore, be more sensitive to changes in temporary social dynamics than stable trait-based characteristics.

Overall, when using a computer-based task and measuring trait levels of narcissism, empathy, extraversion, and agreeableness, we do not support the view that mechanisms underpinning automatic imitation, such as the MNS, systematically vary as a function of stable predispositions to be interested in others or behave in a prosocial manner. However, further research could examine potential relationships between automatic imitation and other stable trait characteristics, which were not measured here.

### Subclinical trait characteristics and automatic imitation

The current study also investigated possible links between automatic imitation and subclinical traits in the typical population. Despite the proposal that autistic-like and schizotypal traits exist on a continuum from subclinical to clinical manifestations [45] and contrary to suggestions that a dysfunctional MNS underpins atypical imitation in ASD and schizophrenia [9,10,61], we found no evidence for a relationship between autistic-like or schizotypal traits and automatic imitation. When we did not control for third variables, schizotypal traits negatively predicted the congruency effect. This relationship, however, was not observed when mean RT and sex were controlled for. These results highlight the methodological importance of controlling for extraneous variables whenever possible [26–28,31,57]. In short, these data are not consistent with the idea that imitation deficits in autism or schizophrenia are due to a dysfunctional MNS.

Two aspects of this result warrant further discussion. First, prior studies that have shown imitation deficits in ASD and schizophrenia have studied intentional rather than automatic imitation [10,40,42,62]. Conversely, in the current task, we measured an RT index of automatic imitation. These different forms of imitation—intentional vs. automatic—are likely to rely on different neurocognitive mechanisms, at least to some extent. As such, our results do not point towards problems in the automatic system in these disorders, but leave open the possibility that some difficulties may arise from systems underpinning intentional imitation.

Second, the present results are consistent with the view that basic matching processes between vision and action may be intact in ASD and schizophrenia but other processes, such as top-down control, may be impaired [5,6,7,8]. Direct evidence for this proposal comes from studies of automatic imitation using an RT paradigm, which shows that individuals with ASD have an intact congruency effect [43,44], but lack modulation of imitation based on social context [33]. Further research could directly investigate whether different forms of imitation (intentional and automatic) are impaired in ASD and schizophrenia, as well as the extent that impairments rely on the MNS or other neurocognitive systems.

### Sex differences in automatic imitation

Although it was not part of our *a prior* predictions, we did find that sex predicted the congruency effect, such that women had a greater tendency to automatically imitate than men. This result could not be explained by sex-differences in stable traits, as we found that no stable trait predicted performance on the imitation task and there were no sex*trait interactions. As such, any pattern of relationship between each trait and automatic imitation was the same for both male and female participants. Furthermore, we found a sex by mean RT interaction that shows that as mean RT becomes slower, the sex difference on the congruency effect becomes larger. This suggests that there is not a general sex difference across all speeds; rather, the sex difference emerges at slower speeds. Two plausible explanations of the sex difference in automatic imitation are outlined below.

First, the result may not represent a sex difference per se, but an own-sex bias. The study was not designed to test for sex differences and, as such, only a female hand was used during the automatic imitation task. Therefore, given the evidence that children copy behaviours more that are demonstrated by same gender models [63] and the strength and ubiquity of ingroup biases even for arbitrarily assigned groups [64], the result could be explained by females showing more sensitivity to an ingroup member than an outgroup member. Second, the result may reflect a basic sex difference in the systems that underpin performance on the automatic imitation task. Based on performance on similar cognitive control tasks, there is currently some limited support for such sex differences. For example, women show larger interference effects than men on flanker [65], oddball [66], as well as gaze- and arrow-cueing tasks [67]. All of these tasks share a similarity with the automatic imitation task: they require inhibition of a response to a task-irrelevant feature in order to enforce a task-relevant response. As such, based on sex differences in the systems that underpin such processes, women may find it more difficult than men to suppress the task-irrelevant stimulus. These proposals, however, remain speculative and further research is required to directly investigate these possibilities.

### Limitations

It is possible that null results could, in part, be due to the accuracy of the underlying measures. For most of our measures, we feel this is an unlikely account of our findings. Cronbach’s alpha analyses demonstrated generally good reliability of the personality measures used. Indeed, only the AQ had suboptimal reliability. Additionally, all measures used, despite being short-form versions, have been previously validated (22,46,48,49,50). Nevertheless, further work that aims to replicate the null effects observed here with more reliable long-form measures of personality are welcomed (e.g., the 50-item AQ).

The imitation task that we used indexes interference produced by both imitative and spatial components of the task. As such, imitation and spatial compatibility between stimulus and response could have contributed to our null findings. In order for this to occur, however, it would require assuming that automatic imitation and spatial compatibility are influenced in opposite directions by variation in personality traits (e.g. someone with more narcissistic tendencies would exhibit high levels of spatial compatibility alongside low levels of automatic imitation). As we have no reason to propose that such a negative relationship exists, we suggest it is an unlikely, yet possible, account of our null findings.

## Conclusion

The present study provides novel insight into the antecedents of automatic imitation. First, we found no relationship between automatic imitation and stable components of personality including empathy and narcissism. As such, we suggest that the relationship between social components of personality and automatic imitation is less universal than initially conceived. Second, we found no relationship between automatic imitation and autistic-like and schizotypal traits, which is not consistent with the view that a dysfunctional MNS underpins atypical imitation abilities in ASD and schizophrenia. Instead, this result suggests that the systems supporting automatic imitation are intact in these disorders and other systems may be responsible for the imitation difficulties observed. More generally, we show the importance for studies of psychological processes to attempt replication experiments [29,30], study larger samples, and control for confounding variables [26–28,31,57]. By doing so, a more accurate estimate of the underlying cogitive architecure is produced.

## Supporting Information

S1 Table Simple correlations between each predictor and the congruency effect for each multiple regression model. (TIFF)
