# Supplementary figures and images for: Investigating the Relationship between Stable Personality Characteristics and Automatic Imitation
Source: PLoS One. 2015 Jun 16;10(6):e0129651. doi: 10.1371/journal.pone.0129651 (PMC4469457; doi:10.1371/journal.pone.0129651)

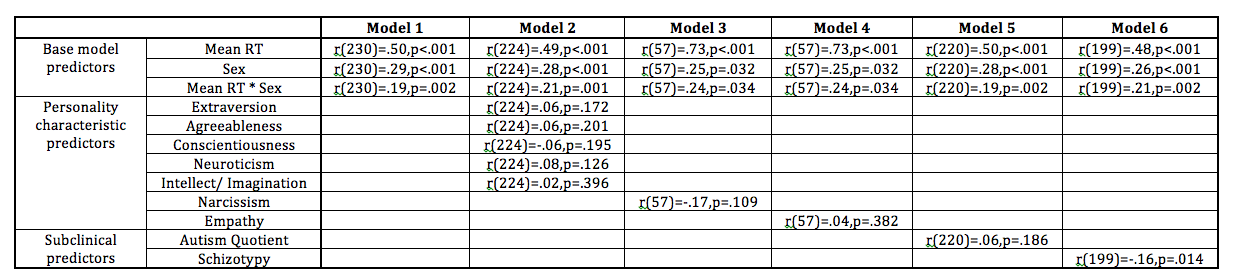

Supplement: S1 Table — (TIFF) [file pone.0129651.s001.tiff]
